# Care of the Teeth

**Published:** 1873-05

**Authors:** D. C. Hawxhurst


### CARE OF THE TEETH.

BY D. C. HAWXHURST

In this article I purpose giving a few brief hints for the management of the teeth. And I shall address my words directly to the patient. In my own practice I have often felt that my advisatory remarks to patients constituted the more important part of the service which I had rendered them. But I have wished that I were able to put a few recorded sentences into their hands which would tend to arouse in them a sense of the importance of the subject.

Among measures for the preservation of the teeth, cleanliness ranks first. It is more important than all things else. You may observe that those surfaces of the teeth which are kept freest from soft deposits of food by friction of the lips and tongue, are also freest from decay ; on the other hand, all confined localities, interstices and fissures, where impurities are sheltered from friction and removal, are burrowed into by decaying cavities. It is often asked: "Why do teeth decay most often in secret and hidden places, making the cavities both hard to find and difficult to fill?" It is because filth accumulates here and rusts out the hardest enamel. You have not done your whole duty by your teeth until you have removed the last atom of impurity from the most sheltered position between the teeth.

As a cleansing agent, the tooth-brush is indispensable; nothing has ever been invented that can equal it. It should not be too stiff, else it will wound the sensitive edges of diseased gums, and become an instrument of destruction to the very organs it is intended to save. It should not be used rashly, but with extremest care, so as not to injure the tender membranes of the gum while exploring the most confined interstices after impurity.

You do not know how to use a tooth-brush by instinct, as a bird builds her nest; it is a thing to be learned. Perhaps you have been using a brush for years while your teeth are still decaying. Go to some dentist, and assure him that you will not be offended by the truth: he will tell you that you do not half use your brush.

The brush should be used perpendicularly; it should be forced between the teeth both back and front; it should be carried briskly over the ends and into the fissures of the double teeth; it should be used on the inside of the arch; it should pursue its relentless search on every side of the wisdom teeth. The tooth-brush should also be used with an abundance of tepid water, else it will only stir up the impurities without removing them. Rinsings of soft water may be required to wholly dissolve and carry them away.

How often should the tooth-brush be used? For most people, so often that it will never get dry from one using to another. The brush must in any case be used as often as you eat. "Twice a week," and oftener when you "go visiting," will not satisfy the demands of hygiene. The organic atoms of food do not long lie exposed to the warmth and moisture of the mouth, without undergoing what is termed putrefactive decomposition. The result of this is an acrid, chemical agent that attacks and slowly decomposes the enamel. There is, therefore, no safety unless the teeth are kept absolutely free from the pulpy remains of food.

You should never eat between meals. Cleansing the teeth is useful only until next you taste food; immediately after *that*, they need a second cleansing. To go about between regular meals, tasting an apple here, eating a raspberry there, nibbling a crumb of biscuit at one time, or a cracker at another, is the high road to ruin for teeth. Such a course is disturbing to the stomach, even though you swallow but *little;* it is destruction to the teeth though you swallow *none.* There is but one sensible and safe course. As often as food enters the mouth, it must be followed by the brush and water, even though it be forty times a day. Will you therefore keep nibbling and rinsing all day? It is better to eat what you need, cleanse well your mouth, and, after that, keep it closed against the smallest morsel. Its purity should be held sacred against the slightest flavor of any aliment until the next meal. In this way *only* can you escape the frightful mutilation of the teeth which is going on in the mouths of those about you. Do you think that you can save your teeth by a less rigid adherence to the laws of dental hygiene? Perhaps; but the chances are wholly *against* you.

Most persons should devote from five to ten minutes each day to cleansing the teeth. Brushes of different quality, tooth-picks, floss silk for crowded teeth, should all be at hand. If you do not know how to use all these things, get some dentist to show you. Havn't time for all this? Very well! You will soon have time to spend with your dentist in having expensive fillings inserted. And when all is done, perhaps he will tell you: "The fillings are warranted if you keep your teeth absolutely clean, not otherwise." If you would save your children much suffering, and shield them from the worst kind of mortification; if you would preserve their bodies pure and perfect, temples of the soul, teach them how to care for their teeth.

